# Supplementary material for: Synergistic antifungal effects of botanical extracts against Candida albicans
Source: PLoS One. 2026 Jan 12;21(1):e0340665. doi: 10.1371/journal.pone.0340665 (PMC12795357; doi:10.1371/journal.pone.0340665)
Supplement: Attachment — Submitted filename: 250804_Reviewer_HY.docx [file pone.0340665.s009.docx]

The manuscript "Synergistic antifungal effects of botanical extracts against *Candida albicans*" is a very interesting manuscript which explained quite detail about the interactions of many active compounds. There are some comments below.

Line 32: *P. granatum* should be written completely *Punica granatum* since it is stated for the first time in the manuscript.

Line 60: Please change word “antibiotic” to “antifungals”.

Line 88: Please verify the species name *Matricularia chamomilla* or *Matricaria chamomilla* (in Table 1).

Table 1: In my opinion, it is better to put Table 1 in Materials and Methods rather than in Introduction.

Line 97: Please write “10 µL” in words since it is in the beginning of a sentence.

Line 136, 138, 141, 142 : Please correct typo “Sigma-Aldritch” to “Sigma-Aldrich”.

Line 146: Please write “200 mg” in words since it is in the beginning of a sentence.

Line 178: “minimum inhibitory concentration” should be written for the first time in Line 80 “lower minimum inhibitory concentration (MIC)”.

Line 191: Please change word “antibiotic” to “antifungal”.

Line 198: Please write “100 µL” in words since it is in the beginning of a sentence.

Table 3: Why was *Humulus lupulus* not tested in checkerboard analysis?. In Line 222-226, it seems that *H. lupulus* was not excluded. It has low MIC_50_ (i.e., 0.625 mg/mL).

Line 236: The lowest FICI score is 0.039 (highly synergistic). Range of FICI is 0.039 to 4.0, based on data shown in Table 3.

Line 241-242: Please verify the econazole concentration as positive control. In Line 241-242: econazole concentration is 2 mg/mL, whereas in Line 191 econazole 30 µg/mL.

Figure 2: Please check again the colour in figure 2 with the data in Table 3, and consistent with the criteria of FICI (i.e., Synergistic = FICI < 0.5, additive = 0.5 ≤ FICI < 1, neutral = 1 ≤ FICI < 2, antagonistic = FICI ≥ 2). Below are some inconsistencies:

- Figure 2A. *H. canadensis*/*P. granatum* MIC_90_ 0.50 is additive (Table 3). Therefore, the colour should be light green.
- Figure 2A. *P. granatum*/*E. globulus* MIC_90_ 1.0 is neutral (Table 3). Therefore, the colour should be pink.
- Figure 2B. *H. canadensis*/*P. amurense* MIC_90_ 0.50 is additive (Table 3). Therefore, the colour should be light green.
- and other cells

Figure 3: Please verify the MIC_50_ of *H. canadensis*. In table 2, MIC_50_ of *H. canadensis* is 2.5 mg/mL. However in Figure 3, in 1 mg/mL *H. canadensis* have >50% inhibition.

Line 348: The lowest FICI value of *H. canadensis* is 0.039, combined with *E. globulus*. Therefore, the value is “0.52 to 0.039”.
