# Supplementary material for: Synergistic antifungal effects of botanical extracts against Candida albicans
Source: PLoS One. 2026 Jan 12;21(1):e0340665. doi: 10.1371/journal.pone.0340665 (PMC12795357; doi:10.1371/journal.pone.0340665)
Supplement: Attachment — Submitted filename: Response to Reviewers.docx [file pone.0340665.s010.docx]

Response to Reviewers

PONE-D-25-38285

Synergistic antifungal effects of botanical extracts against *Candida albicans*

PLOS ONE

October 25, 2025

We thank the Editor and the Reviewers for their careful evaluation of our manuscript and for providing insightful comments that have helped us improve the quality and clarity of our work. Below, we provide a detailed, point-by-point response to all comments. Changes made in the revised manuscript are indicated by tracked changes.

**Summary of Major Revisions:**

- Created new supplementary file with raw data and calculations. S1_File contains raw data for MIC_50_ and MIC_90_ values, S2_File contains raw data for synergy testing MIC_50_ and MIC_90_ values, and S3_File contains raw data for single compound MIC_50_ and MIC_90_.
- Edited various typos and figure components as suggested by reviewer #1, and changed some formatting based on PLOS One guidelines.

**Revised Funding Statement:**

This work was supported by NIH / ODS / NCCIH Botanical Center Grant (P50 AT002776 to IR), and the NJ Agricultural Experiment Station of Rutgers, The State University of NJ. Funding support for KA and JH was provided by the National Center for Complementary and Integrative Health through training grant 5T32AT004094. There was no additional external funding received for this study. The funders had no role in study design, data collection and analysis, decision to publish, or preparation of the manuscript.

**Point-by-point Responses:**

**1. Line 32: *P. granatum* should be written completely *Punica granatum* since it is stated for the first time in the manuscript.**

We have changed the formatting for this as suggested.

**2. Line 60: Please change word “antibiotic” to “antifungals”.**

We have changed the wording as suggested.

**3. Line 88: Please verify the species name *Matricularia chamomilla* or *Matricaria chamomilla* (in Table 1).**

We verify that the species used for this research is *Matricaria chamomilla*, or German chamomile, as written in the manuscript in Table 1, and have changed the typo.

**4. Table 1: In my opinion, it is better to put Table 1 in Materials and Methods rather than in Introduction.**

Thank you for the suggestion, however we believe that Table 1 is best suited for the Introduction as it summarizes previous literature on bioactive compounds in the studied plants and provides context to why they were selected for the synergy testing. It also introduces important conceptual information rather than procedural information.

**5. Line 97: Please write “10 µL” in words since it is in the beginning of a sentence.**

Changed the structure of the sentence.

**6. Line 136, 138, 141, 142 : Please correct typo “Sigma-Aldritch” to “Sigma-Aldrich”.**

Changed as suggested.

**7. Line 146: Please write “200 mg” in words since it is in the beginning of a sentence.**

Changed the structure of the sentence.

**8. Line 178: “minimum inhibitory concentration” should be written for the first time in Line 80 “lower minimum inhibitory concentration (MIC)”.**

Changed as suggested.

**9. Line 191: Please change word “antibiotic” to “antifungal”.**

Changed as suggested.

**10. Line 198: Please write “100 µL” in words since it is in the beginning of a sentence.**

Changed the structure of the sentence.

**11. Table 3: Why was *Humulus lupulus* not tested in checkerboard analysis?. In Line 222-226, it seems that *H. lupulus* was not excluded. It has low MIC_50_ (i.e., 0.625 mg/mL).**

*H. lupulus* was not tested because our criteria for determining extracts for candidates of synergy testing was focused on higher overall percent inhibition. Although *H. lupulus* displayed a low MIC_50,_ on the other hand for MIC_90_, it had overall poorer results. For example, although *H. lupulus* and *P. granatum* are both depicted as MIC_90_ ≥20, at 10 mg/mL *H. lupulus* has only a 50.92% inhibition whereas *P. granatum* has 88.10% inhibition against *C. albicans*. This data in Table 3 does not capture this nuance, but additional supplementary information has been added in S1_File that reflects this.

**Line 236: The lowest FICI score is 0.039 (highly synergistic). Range of FICI is 0.039 to 4.0, based on data shown in Table 3.**

Changed as suggested.

**Line 241-242: Please verify the econazole concentration as positive control. In Line 241-242: econazole concentration is 2 mg/mL, whereas in Line 191 econazole 30 µg/mL.**

Adjusted to fit most recent data collections – 30 µg/mL.

**Figure 2: Please check again the colour in figure 2 with the data in Table 3, and consistent with the criteria of FICI (i.e., Synergistic = FICI < 0.5, additive = 0.5 ≤ FICI < 1, neutral = 1 ≤ FICI < 2, antagonistic = FICI ≥ 2). Below are some inconsistencies:**

- **Figure 2A. *H. canadensis*/*P. granatum* MIC_90_ 0.50 is additive (Table 3). Therefore, the colour should be light green.**
- **Figure 2A. *P. granatum*/*E. globulus* MIC_90_ 1.0 is neutral (Table 3). Therefore, the colour should be pink.**
- **Figure 2B. *H. canadensis*/*P. amurense* MIC_90_ 0.50 is additive (Table 3). Therefore, the colour should be light green.**
- **and other cells**

Changed the ranges on Figure 2 so values at the thresholds (i.e. 0.500, 1.000, etc.) have appropriate colors for their synergy designation.

**Figure 3: Please verify the MIC_50_ of *H. canadensis*. In table 2, MIC_50_ of *H. canadensis* is 2.5 mg/mL. However in Figure 3, in 1 mg/mL *H. canadensis* have >50% inhibition.**

MIC_50_ is 2.5 mg/mL. The original value of % inhibition at 1 mg/mL in Figure 3 was a mistake and has been fixed.

**Line 348: The lowest FICI value of *H. canadensis* is 0.039, combined with *E. globulus*. Therefore, the value is “0.52 to 0.039”.**

Changed as suggested.

**Minor comments:
Please, explain the reason to choose the *C. albicans* strain 10231.**

We chose to use *C. albicans* strain 10231 because it is widely studied and characterized and is a designated strain for QC standards for various assays by CLSI and EUCAST, whose methods were used to base our own study methodology on.
