# Supplementary material for: Synergistic antifungal effects of botanical extracts against Candida albicans
Source: PLoS One. 2026 Jan 12;21(1):e0340665. doi: 10.1371/journal.pone.0340665 (PMC12795357; doi:10.1371/journal.pone.0340665)
Supplement: Attachment — Submitted filename: Response_to_Reviewers_auresp_2.docx [file pone.0340665.s011.docx]

Response to Reviewers

PONE-D-25-38285R1

Synergistic antifungal effects of botanical extracts against *Candida albicans*

PLOS ONE

October 25, 2025

We thank the Editor and the Reviewers for their careful evaluation of our manuscript and for providing insightful comments that have helped us improve the quality and clarity of our work. Below, we provide a detailed, point-by-point response to all comments. Changes made in the revised manuscript are indicated by tracked changes.

**Point-by-point Responses:**

1. **Regarding Table 1, while your intention to summarize prior literature and provide conceptual context is noted, relocation to the Materials and Methods section is recommended, consistent with the reviewer’s suggestion and standard scientific conventions. The Introduction should remain a narrative overview supported by citations, rather than a place for structured data. Placing the table in the Methods section, where the selection of plant species is described, will improve clarity, enhance manuscript flow, and allow for more appropriate referencing in the Results and Discussion when interpreting outcomes related to the extracts.**

Moved Table 1 to the “Plant materials” subsection in Materials and Methods, as recommended, with additional brief context on their selection.

2. **Please include a brief rationale in the Methods section for selecting Candida albicans strain ATCC 10231. This will help clarify its relevance and support reproducibility.**

Provided a brief explanation of ATCC 10231 in “Reagents, chemicals and fungal strain” subsection in Materials and Methods as recommended.
